# Magnetic-field induced multiple topological phases in pyrochlore iridates with Mott criticality

**DOI:** 10.1038/ncomms15515

**Published:** 2017-05-24

**Authors:** Kentaro Ueda, Taekoo Oh, Bohm-Jung Yang, Ryoma Kaneko, Jun Fujioka, Naoto Nagaosa, Yoshinori Tokura

**Affiliations:** 1 Department of Applied Physics, University of Tokyo, Hongo 7-3-1, Bunkyo-ku, Tokyo 113-8656, Japan; 2 Department of Physics and Astronomy, Seoul National University, Seoul 08826, Republic of Korea; 3 Center for Correlated Electron Systems, Institute for Basic Science (IBS), Seoul 08826, Republic of Korea; 4 Center for Theoretical Physics (CTP), Seoul National University, Seoul 08826, Korea; 5 PRESTO, Japan Science and Technology Agency, Kawaguchi, Saitama 332-0012, Japan; 6 RIKEN Center for Emergent Matter Science (CEMS), Wako 351-0198, Japan

## Abstract

The interplay between electron correlation and spin–orbit coupling in solids has been proven to be an abundant gold mine for emergent topological phases. Here we report the results of systematic magnetotransport study on bandwidth-controlled pyrochlore iridates *R*_2_Ir_2_O_7_ near quantum metal-insulator transition (MIT). The application of a magnetic field along [001] crystallographic direction (*H*//[001]) significantly decreases resistivity while producing a unique Hall response, which indicates the emergence of the novel semi-metallic state in the course of the magnetic transformation from all-in all-out (AIAO, 4/0) to 2-in 2-out (2/2) spin configuration. For *H*//[111] that favours 3-in 1-out (3/1) configuration, by contrast, the resistivity exhibits saturation at a relatively high value typical of a semimetal. The observed properties can be identified to reflect the emergence of multiple Weyl semimetal states with varying numbers of Weyl points and line nodes in respective spin configurations. With tuning effective bandwidth, all these states appear to concentrate around the quantum MIT region, which may open a promising venue for topological phenomena and functions.

## Abstract (web summary)

The interplay between multiple electronic interactions in solid promotes the emergence of exotic phases. Here, Ueda *et al*. report magnetotransport study on pyrochlore iridates *R*_2_Ir_2_O_7_ near quantum metal-insulator transition reflecting the emergence of multiple Weyl semimetal states.

The pyrochlore *R*_2_Ir_2_O_7_ is composed of the networks of corner-linked tetrahedra of rare-earth *R* ions and Ir ones. This geometrically frustrated lattice offers a fertile ground to host exotic electronic/magnetic states^1^,^2^,^3^,^4^. Recent angle-resolved photoemission spectroscopy unveils that the *R*=Pr compound is a unique semimetal with a quadratic band crossing at *Γ* point, which is an essential ingredient for versatile topological states^5^; for instance, the antiferromagnetic all-in all-out (AIAO) magnetic order, which breaks time-reversal symmetry while preserving crystal symmetry, lifts the band degeneracy, leading to linearly dispersed band touching points in three dimension, here termed Weyl semimetal (WSM (4/0))^3^,^6^,^7^. Another possibility for unconventional electronic states is intensively discussed with different magnetic patterns^8^,^9^,^10^,^11^. Owing to the uniaxial magnetic anisotropy along the cubic [111] or equivalent directions into the center of the tetrahedron, various magnetic pattern can be achieved under the competition between exchange interactions and external magnetic field^12^. For example, when a magnetic field applied along *H*//[001] (*H*//[111]) is strong enough to overcome the exchange interaction, it turns two (three) magnetic moments point inwards and the other two (one) point outwards of the tetrahedron, forming 2/2 (3/1) configuration.

Another key parameter is a one-electron bandwidth, exemplifying the inverse of the effective electron correlation (*U*)^13^. One can finely tune it by applying hydrostatic pressure^14^,^15^ or substituting *R* site^16^,^17^ that can drive metal-insulator transition (MIT); the Pr compound is a paramagnetic semimetal down to 120 mK^18^, whereas the paramagnetic or antiferromagnetic AIAO insulating phase shows up with smaller *R* ionic radius^19^,^20^,^21^, seemingly akin to the correlation-induced MIT as widely observed for 3*d*-electron materials^15^,^22^,^23^. On the verge of quantum MIT (in between *R*=Nd and Pr), however, the unconventional magnetotransport phenomena have been reported, including anomalous Hall effect^24^,^25^, highly metallic AIAO domain walls^26^,^27^ and field-induced MIT^10^,^11^, which may be potentially correlated to the predicted topological states. The quantum MIT involving such correlated topological states may provide an ideal platform of a novel quantum criticality^28^,^29^, but has been rarely explored so far. To address this issue, we perform systematic magneto-transport measurements on *R*=Nd and its partially Pr-substituted (*R*=Nd_0.5_Pr_0.5_) compounds under external pressures (*P*) and magnetic fields (*H*), which allow us to finely and precisely tune the effective bandwidth and magnetic configuration. We have revealed the rich topological phases as a function of bandwidth and magnetic field around the quantum critical point.

## Results

### Electronic/magnetic phase diagram for pyrochlore iridates

**Figure 1:**
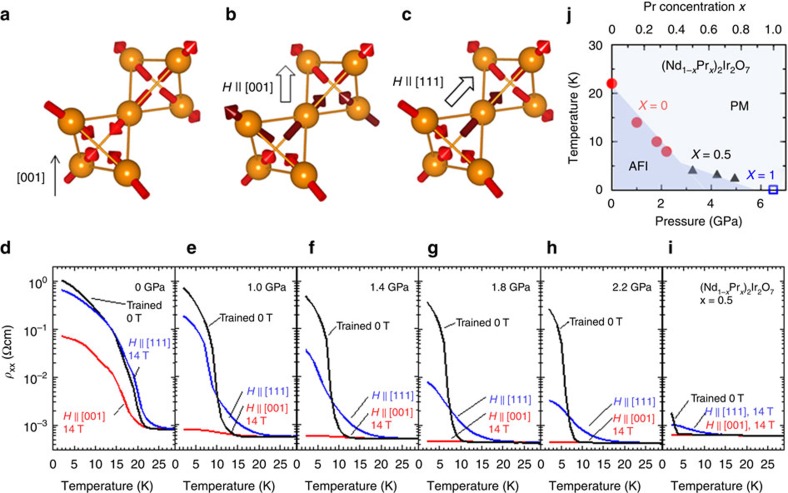
Temperature dependence of resistivity in bandwidth-controlled *R*_2_Ir_2_O_7_. Schematic magnetic configuration for (**a**) all-in all-out state, (**b**) 2-in 2-out state and (**c**) 3-in 1-out state, respectively. Temperature dependence of resistivity for *R*=Nd (*x*=0) at (**d**) 0 GPa, (**e**) 1.0 GPa, (**f**) 1.4 GPa, (**g**) 1.8 GPa, (**h**) 2.2 GPa and (**i**) *x*=0.5 for *R*=Nd_1−*x*_Pr_*x*_ (effectively 3.3 GPa), respectively. The black curves denote the resistivity of trained state which was measured on elevating temperature process at 0 T after magnetic field cooling of 14 T along [111] crystallographic direction, to eliminate the contribution from the metallic magnetic domain walls. The blue lines denote the resistivity under a magnetic field of 14 T applied parallel to [111] direction and the red ones are for a field along [001] direction. (**j**) Metal-insulator transition temperature as a function of pressure (bottom axis) and Pr concentration *x* (top axis). Δ*x*=0.1 corresponds to Δ*P*=0.65 GPa. The circles denote the transition temperature for *x*=0, the triangles are that for *x*=0.5 and the square is that for *x*=1.

We show the temperature dependence of resistivity at several pressures in Fig. 1d–h. The resistivity at ambient pressure increases rapidly below the transition temperature *T*_N_=22 K, which is higher than that of the previous study thanks to the recent improvement of the sample quality (see Methods). The transition temperature systematically shifts to lower temperature with increasing pressure as observed also in previous studies^14^,^15^. Figure 1i displays the temperature dependence of resistivity for the mixed-crystal compound of *x*=0.5 (*R*=Nd_1−*x*_Pr_*x*_), which also shows a sharp increase below 4 K. We plot the *T*_N_ as a function of pressure and chemical substitution *x* in Fig. 1j, using the established empirical relation between the chemical and physical pressures^15^ that the composition change Δ*x*=0.1 corresponds to the pressure change Δ*P=*0.65 GPa; hereafter, we regard *x*=0.5 as being equivalent to the application of *P*=3.3 GPa on *x*=0. The *T*_N_ is almost linearly suppressed as (chemical) pressure increases, enabling us to explore a broad range of effective bandwidth-control effect. It should be noted that the AIAO insulating phase persists up to *P*∼5.0 GPa (*P*∼1.7 GPa on the x=0.5 compound) as shown in the pressure dependence of resistivity for *x*=0.5 (Supplementary Fig. 1). Such robustness of the insulating phase is also reported in ref. 14.

### Anomalous magnetotransport phenomena near MIT

Figure 1d–i also display the resistivity under a magnetic field of 14 T along [001] direction and [111] direction. For *H*//[001], whereas the resistivity slightly decreases by the application of magnetic field at ambient pressure, the abrupt increase of the resistivity below *T*_N_ is significantly suppressed above *P*=1.0 GPa. It means that the systematic application of pressure brings the system to the critical region in which various electronic or magnetic phases strongly compete with each other, as observed for the colossal magnetoresistance in perovskite manganites^30^. It is noteworthy that the similar large magnetoresistance was reported in refs 10, 11 even at ambient pressure. This can be ascribed to the slight off-stoichiometry of the crystal such as iridium deficiency, which somewhat changes the band filling of the system and effectively drive the system closer to the critical region. The applied magnetic field *H*//[111], on the other hand, induces distinct magnetotransport properties from the case of *H*//[001]; the resistivity starts to rise gradually even above *T*_N_ and appears to nearly saturate at lower temperatures. The observed property for each field direction is attributable to the emergence of a novel electronic state induced by *H*//[001] (*H*//[111]), which favours the 2/2 (3/1) magnetic configuration in *R* 4*f* moments as depicted in Fig. 1b,c. In fact, the saturated values of magnetization for *H*//[001] (*H*//[111]) agree well with the expected values in 2/2 (3/1) state (Supplementary Fig. 2a–d). The no *d*-electron analog Nd_2_Zr_2_O_7_, in which the Nd 4*f* moment forms AIAO magnetic order at zero field, also shows magnetic field-induced 2/2 or 3/1 order^31^. Furthermore, for the *x*=0.5 compound, the peak of the specific heat divided by temperature gradually shifts to higher temperature, while being broadened on increasing *H*//[001] (*H*//[111]) (Supplementary Fig. 2e,f); these features clearly indicate that the increasing magnetic field *H*//[001] (*H*//[111]) induces the 2/2 (3/1) type magnetic order at higher temperatures than *T*_N_^32^,^33^. Owing to the magnetic coupling between 4*f* and 5*d* moments, the magnetic structure of 5*d* moments can follow that of 4*f* ones, leading to the observed transport properties. It is to be noticed that the magnetic field is always applied perpendicular to the electric current in this experiment (see Methods), which excludes the possibility of chiral anomaly effect, that is, the negative magnetoresistance effect with the current parallel to the magnetic field, recently observed in a WSM material^34^. Hence, the observed anisotropic magnetoresistance genuinely stems from the modulation of the magnetic configuration.

### Magnetotransport properties for H//[001]

**Figure 2:**
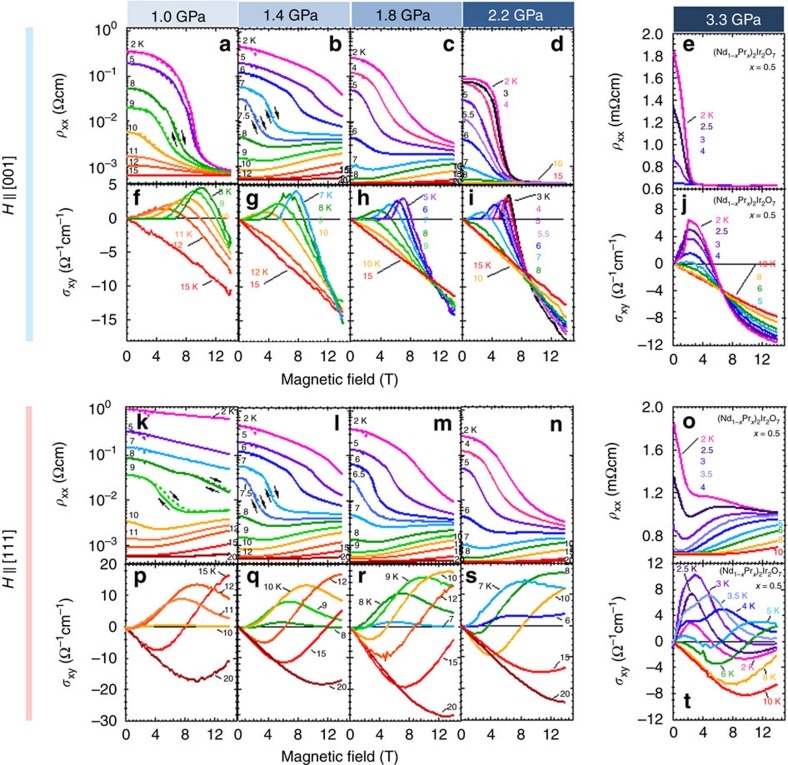
Magnetotransport properties of (Nd_1−*x*_Pr_*x*_)_2_Ir_2_O_7_ at several pressures. Magnetic field dependence of resistivity (**a**–**e**) and Hall conductivity (**f**–**j**) for a field along the [001] crystallographic direction at (**a**,**f**) 1.0 GPa, (**b**,**g**) 1.4 GPa, (**c**,**h**) 1.8 GPa, (**d**,**i**) 2.2 GPa and (**e**,**j**) 3.3 GPa (*x*=0.5), respectively. Magnetic field dependence of resistivity (**k**–**o**) and Hall conductivity (**p**–**t**) for a field along [111] direction at (**k**,**p**) 1.0 GPa, (**l**,**q**) 1.4 GPa, (**m**,**r**) 1.8 GPa, (**n**,**s**) 2.2 GPa and (**o**,**t**) 3.3 GPa (*x*=0.5), respectively. The solid (broken) lines are the resistivity on increasing (decreasing) field process which is indicated by black arrows.

**Figure 3:**
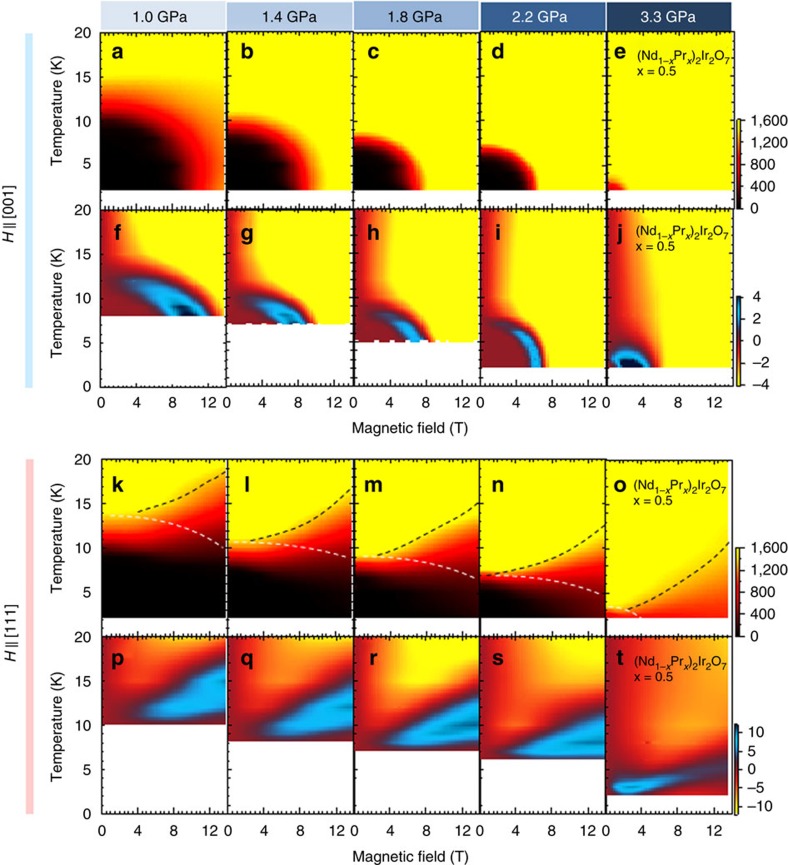
Contour plot of conductivity and Hall conductivity. Contour map of conductivity (**a**–**e**) and Hall conductivity (**f**–**j**) for a field along [001] direction in the plane of temperature and magnetic field at (**a**,**f**) 1.0 GPa, (**b**,**g**) 1.4 GPa, (**c**,**h**) 1.8 GPa, (**d**,**i**) 2.2 GPa and (**e**,**j**) 3.3 GPa (*x*=0.5), respectively. Contour plot of conductivity (**k**–**o**) and Hall conductivity (**p**–**t**) for a field along [111] at (**k**,**p**) 1.0 GPa, (**l**,**q**) 1.4 GPa, (**m**,**r**) 1.8 GPa, (**n**,**s**) 2.2 GPa and (**o**,**t**) 3.3 GPa (*x*=0.5), respectively. The black (white) broken lines are the guide to the eyes for the crossover between paramagnetic metal and 3-in 1-out semimetal state (all-in all-out insulator state and 3-in 1-out semimetal state). The colour bars in figures (**e**,**j**,**o**,**t**) denote the longitudinal conductivity *σ*_*xx*_ (Ω^−1^ cm^−1^) (Hall conductivity *σ*_*xy*_ (Ω^−1^ cm^−1^)), respectively.

The magnetic field dependence of resistivity for *H*//[001] at several pressures are given in the top panels of Fig. 2. The sharp decrease of resistivity is accompanied by a hysteresis between field-increasing and field-decreasing processes below *T*_N_, as discerned in previous studies^10^,^11^. The Hall conductivity shown in Fig. 2f–j provides important insights into the observed field-induced MIT. Above *T*_N_, the Hall conductivity is nearly proportional to magnetic field, typical of normal Hall effect. By contrast, below *T*_N_, the Hall conductivity exhibits non-monotonous field dependence; it is nearly zero at low magnetic fields, abruptly rises up at intermediate fields and eventually decreases towards a negative value at high fields. This feature is more pronounced as temperature is decreased. A similar sign change of Hall response is also observed in the Nd_2_Ir_2_O_7_ polycrystals^25^. The observed complexity of the Hall response can be hardly explained in terms of the conventional normal or anomalous Hall resistivity^35^. The contour plots of the longitudinal and Hall conductivity in the plane of temperature and magnetic field for *H*//[001] at various pressures are shown in Fig. 3a–j, respectively.

**Figure 4:**
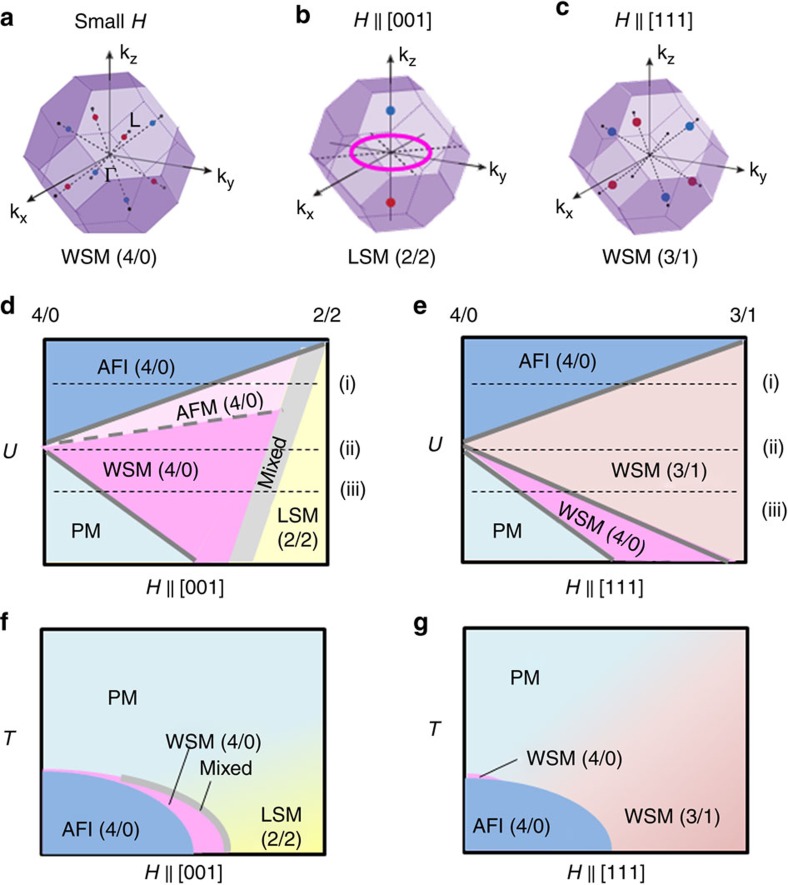
Schematic band structure and phase diagram. Schematic picture of the distribution of Weyl points and line nodes in the three-dimensional momentum space for (**a**) WSM with AIAO state (4/0) in a small magnetic field, (**b**) LSM with 2-in 2-out state (2/2) in a magnetic field along [001] direction (*H*//[001]), and (**c**) WSM with 3-in 1-out state (3/1) in a field along [111] direction (*H*//[111]), respectively. Red (blue) points denote the Weyl points with positive (negative) sign of the charge chirality and a purple ring denotes the line node. Schematic phase diagrams obtained from the mean-field lattice model and the low energy effective *k*·*p* Hamiltonian, where the vertical axis is the electron correlation *U* and the horizontal axis is a magnetic field along (**d**) *H*//[001] and (**e**) *H*//[111]. AFI and PM stand for antiferromagnetic insulator and paramagnetic metal, respectively. AFM(4/0) denotes an antiferromagnetic metal with electron/hole pockets, which is obtained by smooth deformation of the AFI(4/0) band structure without any band crossing between the conduction and valence bands. ‘Mixed' indicates the phase in which the nodal points of WSM(4/0) and the nodal points/line of LSM(2/2) coexist. The dashed lines (i), (ii) and (iii) denote the transition through variation of magnetic modulation at several *U*. Schematic phase diagram in the plane of temperature and magnetic field along (**f**) *H*//[001] and (**g**) *H*//[111] for the case (i), obtained mainly from the experimental results of magnetotransport (Fig. 3) combined with the assignments of the respective electronic phases (**d**,**e**).

In general, the Hall conductivity is sensitive to the relaxation time. For instance, the vanishing Hall conductivity at low fields reflects the localized nature of electrons, in accord with the relatively large value of resistivity (*ρ*_*xx*_>10 mΩcm). At high fields, on the other hand, the Hall conductivity largely decreases towards a negative value, while the resistivity nearly saturates around *ρ*_*xx*_∼0.4 mΩcm (Fig. 2a–e); one plausible candidate of the electronic phase for this metallic state can be the topological state in the 2/2 configuration, which possesses a nodal line in the *k*_z_=0 plane and two Weyl points on the *k*_z_ axis as presented in Fig. 4b, dubbed here line node semimetal (LSM) following the previous study^10^. The major result of Hall response presented here is a sizable signal with positive sign in an intermediate field region. On increasing field, the Hall conductivity shows a dramatic change including even a sign reversal, which can be attributed to the crossover between the 4/0 WSM (Fig. 4a) and 2/2 LSM (Fig. 4b), as schematically shown in Fig. 4d,f. As 4/0 WSM and 2/2 LSM have different Fermi surface topology, the transition between them requires a significant modification of the band structure near the Fermi level such as accompanied by emerging electron/hole pockets, which can strongly modify the Hall conductivity including its sign changes. Such competing contribution of the normal and anomalous components to the total Hall conductivity are also theoretically calculated shown in Supplementary Fig. 3, which demonstrates the nonmonotonic magnetic field dependency.

In the contour plots of longitudinal and Hall conductivity shown in Fig. 3a–j, we can unveil the characteristic relation between the observed MIT and Hall conductivity for *H*//[001]. Both longitudinal and Hall conductivity are relatively small in a low-field and low-temperature region (AIAO insulating phase). On increasing field, the Hall conductivity shows a dramatic change with a sign reversal, which can be attributed to the crossover between the WSM and LSM, as schematically shown in Fig. 4d,f. Interestingly, the WSM phase, which was theoretically predicted to exist in quite a narrow temperature window at zero field^6^,^7^ and hence would be difficult to detect such an electronic band state by optical^36^ and angle-resolved photoemission spectroscopy^37^, can be extended by an application of magnetic field along [001], which deforms the regular 4/0 spin configuration. Moreover, as the pressure increases, both AIAO insulating phase and WSM one appear to shrink, whereas the LSM extends towards zero temperature and zero field. At the quantum critical point, the various competing phases, not only antiferromagnetic Mott insulator and paramagnetic semimetal but also the topological pseudo-4/0 WSM and 2/2 LSM, come close to each other in free energy, apparently merging into the quantum critical point.

### Unconventional semimetal phases in H//[111]

We now turn to the magnetotransport properties for *H*//[111], which are shown in Fig. 2k–t. Right above *T*_N_, the resistivity is largely enhanced by an applied field, whereas the Hall conductivity shows a sharp upturn and changes its sign in high magnetic field (Fig. 2p–t); similar magnetotransport properties are also reported for the paramagnetic *R*=Pr compound at a much lower temperature (*T*=30 mK)^38^. On lowering temperature below *T*_N_, a sharp dip structure is observed around *μ*_0_*H*=3 T in resistivity, attributable to the emergence of metallic domain walls as demonstrated in the previous studies^26^,^27^. More importantly, the resistivity exhibits the unique magnetic field dependence accompanied by a hysteresis between field-increasing and -decreasing processes, which is most pronounced at *T*=9 K and *P*=1.0 GPa as shown in Fig. 2k. Furthermore, the resistivity appears to saturate around *ρ*_*xx*_∼7 mΩcm above *μ*_0_*H*=9 T at which the hysteresis loop closes, indicative of a transition from the 4/0 to the 3/1 magnetic state. To see the evolution of the respective phases more clearly, we plot the contour map of longitudinal conductivity and Hall conductivity in Fig. 3k–t, respectively, and its schematic phase diagram in Fig. 4e,g. One can see that the conductivity is relatively small in a high field region where the sign of Hall conductivity is positive, which can be assigned to the emergence of the new semimetal state with the 3/1 magnetic configuration.

To elucidate the electronic band structure in the 3/1 state, we perform a mean-field calculation (see Methods). The important feature in the 3/1 state is that there is only one trigonal axis parallel to *H*//[111], contrary to the 4/0 state with four trigonal axes. It is noteworthy that a pair of Weyl points are always on one of the four trigonal axes in the AIAO state. As each pair of Weyl points is constrained to be on a one-dimensional line, pair-annihilation can be easily achieved by increasing the pair separation until they merge at the Brillouin zone boundary. In the 3/1 state, however, broken threefold rotation symmetry allows six Weyl points to be shifted away from the relevant one-dimensional subspace and, instead, to move in two-dimensional mirror plane. Whereas the remaining two Weyl points on the trigonal axis parallel to *H* are still constrained, their pair-annihilation results in another WSM with six Weyl points (termed here WSM (3/1)) as described in Fig. 4c. Considering that the point nodes moving in two-dimensional space have smaller collision probability than those moving in one-dimensional space, it is natural to expect that WSM (3/1) is more stable than WSM (4/0), and hence occupies a wider range in the phase diagram; WSM (3/1) phase survives all the way as *H* increases, whereas it is stable within a finite window as *U* increases.

By combining the systematic transport experiments with the theoretical calculations, we suggest that multiple topological states can show up as a function of effective bandwidth (or effective electron correlation *U*) and magnetic field, as schematically shown in Fig. 4d–f. Future neutron and X-ray experiments on the magnetic states as done in refs 19, 20, 21 will serve to verify the present interpretation for the field-induced emergent topological states. Another important feature revealed here is that all these topological states appear to merge towards the magnetic quantum critical point; this may enable the further exploration for new topological quantum states and related exotic electromagnetic responses in this ideal system endowed with Mott criticality.

## Methods

### Single crystal growth

Single crystals of Nd_2_Ir_2_O_7_ and its partially Pr-replaced (Nd_1−*x*_Pr_*x*_)_2_Ir_2_O_7_ were grown by the KF flux method as described in ref. 39. Initially, polycrystalline samples of them were prepared by solid state reactions of rare-earth oxides (Nd_2_O_3_ and Pr_6_O_11_) and iridate IrO_2_. The materials with the prescribed molar ratios were ground, pressed into pellet, and then sintered at 1,273 K for several days. After taken out from the furnace, the polycrystals were ground again and mixed with KF flux in a ratio of 1:200. The mixtures are placed in a platinum crucible covered with a lid. The crucible was cooled down to 1,123 K at a rate of 2 K h^−1^ following anneal for 3–5 h at 1,373 K. After cooling, crystals were separated from the KF residual flux by rinsing it out with distilled water. Octahedral-shaped single crystals were obtained as reported in ref. 39. The crystals were characterized by x-ray diffraction. The qualities of the present samples are improved and the transition temperature becomes higher than that of the crystals previously reported in ref. 10.

### Transport and specific heat measurements

Transport, magnetization and specific heat measurements were performed using Physical Property Measurement System (Quantum Design). Resistivity (Hall conductivity) was measured by a standard four-probe method with the current direction parallel to [110] crystalline direction while the magnetic field along both [001] and [111] crystallographic directions was applied perpendicular to the current. The Hall conductivity is deduced by the anti-symmetrization of the raw transverse signals perpendicular to the electric current. Pressure was generated by a piston-cylinder pressure cell for Physical Property Measurement System. To keep the samples in a hydrostatic pressure, Daphne 7474 oil was used as the pressure-transmitting medium. Pressure was determined by examining the superconducting transition temperature of lead.

### Theoretical analysis

To understand the magneto-transport experiment, we first performed a numerical study of the lattice Hamiltonian^8^, 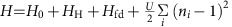 where 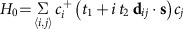
+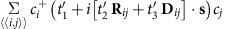. Here *t*_1,2_ 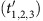 indicates the hopping amplitude between nearest-neighbour (next nearest neighbour) Ir sites, and the Pauli matrices s_*x,y,z*_ represent the doublets with the total angular momentum *J*_eff_=1/2. The real vectors **d**_*ij*_, **R**_*ij*_, **D**_*ij*_ describe **s** dependent hopping terms. 
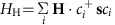
 denotes Zeeman coupling to external magnetic field **H**. *H*_fd_ indicates the *f*–*d* exchange coupling between Ir and rare-earth moments, which has the following form 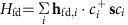 where **h**_fd,*i*_ indicates the effective magnetic field at the *i*-th Ir site due to six neighbouring rare-earth spins around it. Here we treat each rare-earth spin as an Ising spin aligned along its local trigonal axis. Finally, the last term describes electron correlation effect due to the local Hubbard-type interaction (*U*) between Ir electrons. To treat the Coulomb interaction, we employ a mean field approximation by introducing local order parameters *m*_1,2,3,4_ at each site *i*=1, 2, 3, 4 in a unit cell. To facilitate the analysis, we assumed that the main role of the *f*–*d* exchange and the external magnetic field is to rotate the Ir spin orientation from the AIAO to 2-in 2-out (or 3-in 1-out) state when *H*//[001] (*H*//[111]). Then, we can examine the band structure by changing the direction of Ir moments continuously for a given magnitude of Ir moments.

To confirm the results from the lattice Hamiltonian analysis, we also performed the effective model analysis by constructing low energy Hamiltonian near *Γ* or *L* points. For *H*//[001], the results from the lattice Hamiltonian study are already reported in ref. 10. Here we performed additional low-energy Hamiltonian analysis near *Γ* point and obtained consistent results. Namely, magnetic field-induced modulation of Ir spin orientation induces a WSM with point nodes and also an LSM with a line node (LSM) accompanying two additional point nodes. On the other hand, when *H*//[111], we found the transition from a WSM (4/0) with eight Weyl points to a WSM (3/1) with six Weyl points numerically. To support this, we provide detailed effective Hamiltonian analysis as shown in the following.

As a pair annihilation of Weyl points occurs at an *L* point, we need an effective Hamiltonian constructed near the *L* point. In the absence of magnetic field *H*//[111], the Hamiltonian at the *L* point is invariant under inversion (*P*), a combination of a mirror and time reversal (MT), and a threefold rotation about *Γ–L* (C_3_). One can find that *P*, MT and C_3_ can be represented by *P*=*σ*_*z*_ , MT=*K*, C_3_=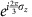 where *σ*_*x*,*y*,*z*_ denotes the two bands touching at the *L* point and *K* stands for complex conjugation. Then the effective Hamiltonian near the *L* point can generally be written as 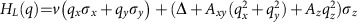 where *v*, Δ, *A*_*xy*_, *A*_*z*_ are constants and *q*_z_ is the momentum along the *Γ*–*L* direction. This Hamiltonian describes a WSM (a gapped insulator) when 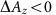 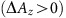. In the presence of *H*//[111], the Weyl points can be separated into two groups. First, in the case of the Weyl point pair located parallel to *H*//[111], P, MT and C_3_ symmetries are all preserved, thus the relevant Hamiltonian maintains the same form as above, except the fact that the constants *v*, Δ, *A*_*xy*_ and *A*_*z*_ depend on *H*. For example, 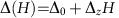. Thus, the magnetic field *H* can control the transition between a WSM and a gapped insulator. On the other hand, in the case of the other six Weyl points, the relevant effective Hamiltonian has a more complicated form since C_3_ symmetry is broken and the magnetic field is not along the local *z* axis. Assuming 
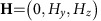
, the effective Hamiltonian becomes 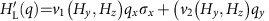 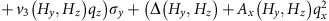 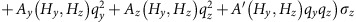 where 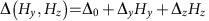 and other constant terms have similar structure. One can clearly see that the location of Weyl points is no longer on the *Γ–L* direction (or the local *z* direction) due to the magnetic field. Although a pair annihilation of Weyl points can also occur in principle, the comparison to the tight-binding analysis shows that, in general, the magnetic field shifts the location of Weyl points away from the *L* point stabilizing the WSM phase whereas the electron correlation forces the Weyl points to move towards the *L* point inducing the transition to a gapped insulator.

### Data availability

The data that support the findings of this study are available from the corresponding author upon reasonable request.

## Additional information

**How to cite this article:** Ueda, K. *et al*. Magnetic-field induced multiple topological phases in pyrochlore iridates with Mott criticality. *Nat. Commun.* **8**, 15515 doi: 10.1038/ncomms15515 (2017).

**Publisher's note**: Springer Nature remains neutral with regard to jurisdictional claims in published maps and institutional affiliations.

## Supplementary Material

Supplementary Information Supplementary Figures and Supplementary References

Peer Review File
