# Supplementary material for: Magnetic-field induced multiple topological phases in pyrochlore iridates with Mott criticality
Source: Nat Commun. 2017 May 24;8:15515. doi: 10.1038/ncomms15515 (PMC5458080; doi:10.1038/ncomms15515)
Supplement: Supplementary Information — Supplementary Figures and Supplementary References [file ncomms15515-s1.pdf]

Supplementary Figure 1: The temperature dependence of resistivity for *x*=0.5 compound at several pressures. As pressure increases, the steep upturn of resistivity gradually shifts to lower temperature down to 2.7 K at 1.7 GPa. It means that the antiferromagnetic insulating phase persists in the present region of low temperature and high pressure, in accord with the previous study[1,2].

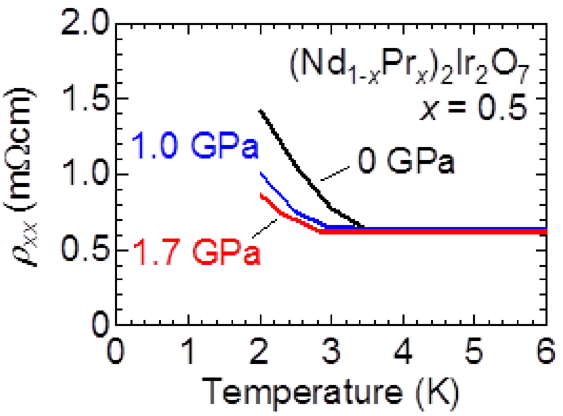

Supplementary Figure 2: The magnetic field dependence of magnetization and specific heat.

Magnetic field dependence of magnetization for x=0 ((a) and (b)) and x=0.5 ((c) and (d)) compound (R=$Nd_{1-x}Pr_x$) at several temperatures in a magnetic field along (a),(c) [001] crystallographic direction and (b),(d) [111] direction, respectively. Temperature dependence of specific heat divided by temperature (C/T) for x=0.5 compound in a magnetic field along (c) [001] direction and (d) [111] direction, respectively. The dashed gray line denotes the C/T of R = Eu polycrystal (with nonmagnetic R ions) to indicate the lattice contribution. As magnetic field increases, the magnetization monotonically increases and saturates in high field. The saturated value is about 1.4 (1.2) $\mu_B/R IrO_{3.5}$, which is in good agreement with the estimated value in 2-in 2-out (3-in 1-out) R 4f magnetic configuration. We show the temperature dependence of specific heat divided by temperature (C/T) for H//[001] in Supplementary Fig. 2e and H||[111] in Supplementary Fig. 2f, respectively. At zero magnetic field, the sharp peak structure is discerned below T_N in C/T, which is a common feature of thermal metal-insulator transition shared with other $R_2Ir_2O_7$. The temperature of the peak in C/T shifts to higher temperature with increasing magnetic field for both H//[001] and H//[111]. Simultaneously, the peak is broadened and its tail extends to ~ 30 K at 14 T. The increasing 2/2 (3/1) type magnetic ordering temperature of R 4f moments by external magnetic field along H//[001] (H//[111]) is commonly observed also in the canonical cases of $Dy_2Ti_2O_7$[3] and $Nd_2Mo_2O_7$[4].

These observed properties indicate that application of a magnetic field induces the magnetic order of R 4f moment even above T_N, leading to the observed transport phenomena as discussed in the main text.

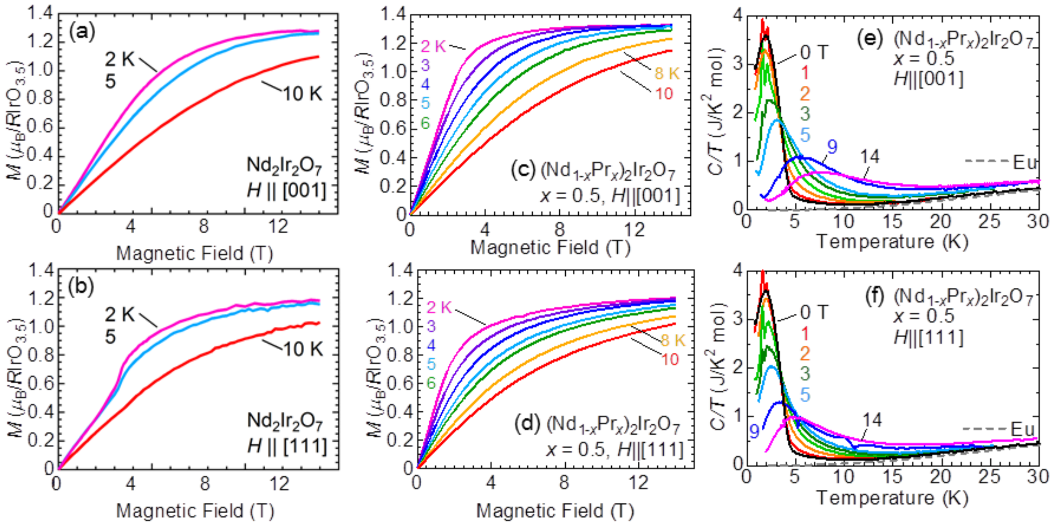
(a)
2 K
5
10 K
Nd2Ir2O7
H || [001]
M (μB/RIrO3.5)
Magnetic Field (T)
(b)
2 K
5
10 K
Nd2Ir2O7
H || [111]
(c) (Nd1-xPrx)2Ir2O7
x = 0.5, H||[001]
2 K
3
4
5
6
8 K
10
(d) (Nd1-xPrx)2Ir2O7
x = 0.5, H||[111]
(e) (Nd1-xPrx)2Ir2O7
x = 0.5
H||[001]
0 T
1
2
3
5
9
14
Eu
C/T (J/K2 mol)
Temperature (K)
(f) (Nd1-xPrx)2Ir2O7
x = 0.5
H||[111]

Supplementary Figure 3: Theoretical calculations for Hall conductivity. Hall conductivity, when the temperature is at 1 Kelvin, obtained by numerically solving the tight binding Hamiltonian described in Methods. Here the grey (orange) line indicates the anomalous (normal) Hall conductivity, and the blue line indicates the summation of the anomalous and normal components. The normal Hall component is computed by using Boltzmann transport theory with a constant transport scattering time about 1 fs which is a reasonable value consistent with the experimental data. In each panel, the horizontal axis indicates external magnetic field (*H*) which continuously deforms the rare-earth spin configuration from 4/0 to 2/2 (from 4/0 to 3/1) when *H*//[001] (*H*//[111]). The magnetic field is scaled relative to the saturation magnetic field (H_{sat}) at which the magnetization reaches its saturated value. The relevant change of the phase diagram is also displayed in each panel. Here the Hall conductivity σ_{xy} is measured in unit of $e^2/(ha_z)$ where a_z is the lattice constant along the z direction. (a) When *H*//[111] starting from the WSM (4/0) at *H*=0 along the dashed line (ii) in Fig. 4e. (b) When *H*//[111] starting from the AFI (4/0) at *H*=0 along the dashed line (i) in Fig. 4e. (c) When *H*|//[001] starting from the WSM (4/0) at *H*=0 along the dashed line (ii) in Fig. 4d. (d) When *H*//[001] starting from the AFI (4/0) at *H*=0 along the dashed line (i) in Fig. 4d. Here LSM (2/2) (WSM (3/1)) indicates the semimetal phase associated with the 2/2 (3/1) rare-earth spin configuration, which has distinct nodal point distribution as compared to WSM (4/0) as explained in the main text. In the panel (c) and (d), Mixed indicates the phase in which the nodal points of WSM(4/0) and the nodal points/line of LSM(2/2) coexist, and AFM(4/0) denotes an antiferromagnetic metal with electron/hole pockets obtained by continuous deformation of the AFI(4/0) band structure without any band crossing between the conduction/valence bands.

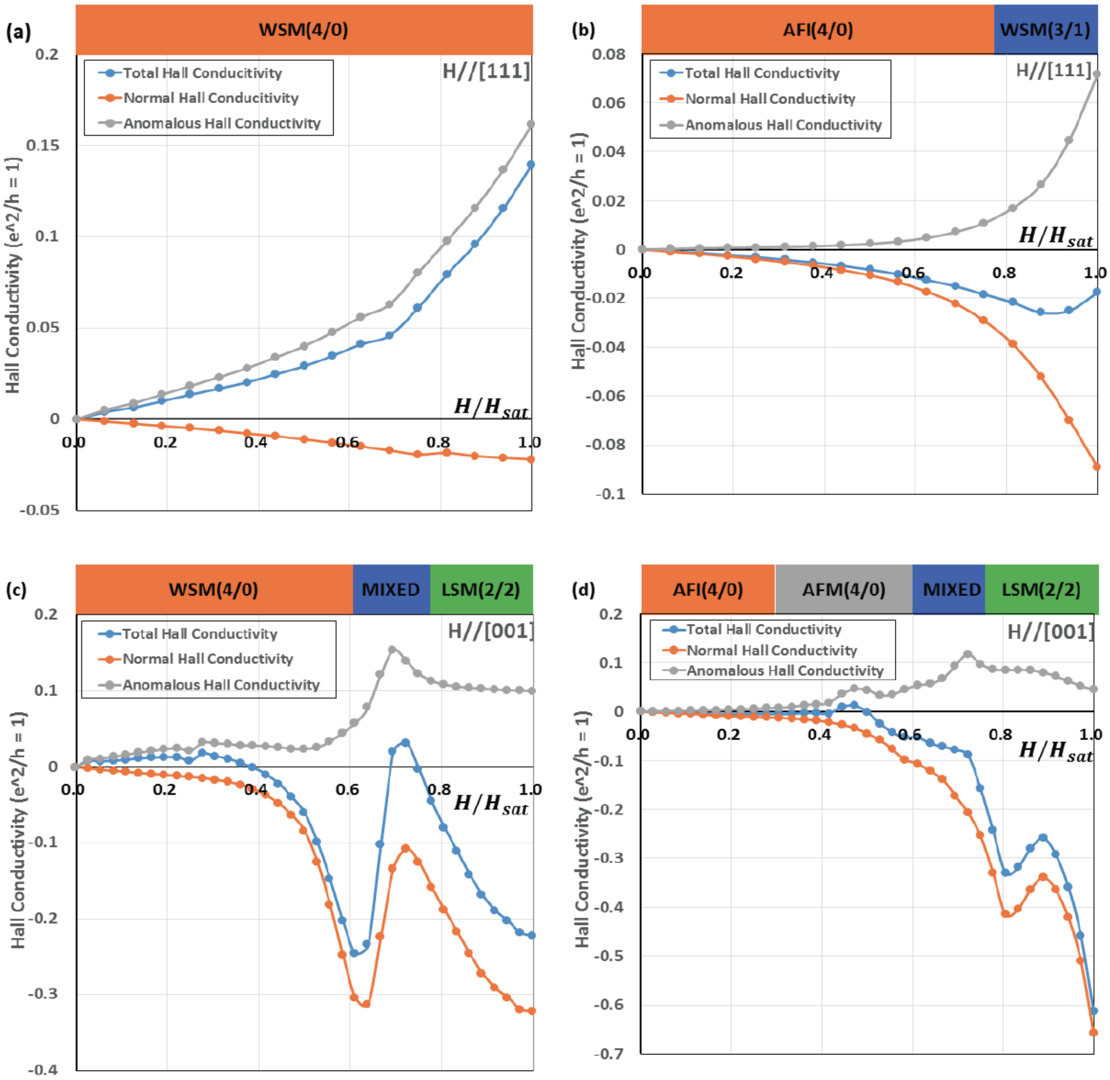

(a)
WSM(4/0)
H//[111]
Total Hall Conductivity
Normal Hall Conductivity
Anomalous Hall Conductivity
Hall Conductivity (e^2/h = 1)
H/H_sat
(b)
AFI(4/0)
WSM(3/1)
H//[111]
(c)
WSM(4/0)
MIXED
LSM(2/2)
H//[001]
(d)
AFI(4/0)
AFM(4/0)
MIXED
LSM(2/2)
H//[001]

Supplementary References

[1] Sakata, M. *et al.* Suppression of metal-insulator transition at high pressure and pressure-induced magnetic ordering in pyrochlore oxide $Nd_2Ir_2O_7$. *Phys. Rev. B* **83**, 041102 (2011).

[2] Ueda, K., Fujioka, J., Terakura, C. & Tokura, Y. Pressure and magnetic field effects on metal-insulator transitions of bulk and domain wall states in pyrochlore iridates. *Phys. Rev. B* **92**, 121110 (2015).

[3] Hiroi, Z., Matsuhira, K., Takagi, S., Tayama, T. & Sakakibara, T. Specific heat of Kagome ice in the pyrochlore oxide $Dy_2Ti_2O_7$. *J. Phys. Soc. Jpn.* **72**, 411 (2003).

[4] Onose, Y., Taguchi, Y., Ito, T. & Tokura, Y. Specific-heat study of the spin-structural change in pyrochlore $Nd_2Mo_2O_7$. *Phys. Rev. B* **70**, 060401 (2004).
