# Supplementary material for: Magnetic-field induced multiple topological phases in pyrochlore iridates with Mott criticality
Source: Nat Commun. 2017 May 24;8:15515. doi: 10.1038/ncomms15515 (PMC5458080; doi:10.1038/ncomms15515)
Supplement: Peer Review File [file ncomms15515-s2.pdf]

**Editorial Note:** this manuscript has been previously reviewed at another journal that is not operating a transparent peer review scheme. This document only contains reviewer comments and rebuttal letters for versions considered at Nature Communications.
